# Comparison of Extracted Teeth and Simulated Resin Blocks on Apical Canal Transportation

**Published:** 2008-10-01

**Authors:** Zohreh Khalilak, Arjang Fallahdoost, Bahareh Dadresanfar, Gholamreza Rezvani

**Affiliations:** 1 *Department of Endodontics, Dental School, Islamic Azad University/ Iranian Center for Endodontic Research, Tehran, Iran*; 2 *General practitioner, Tehran, Iran*

**Keywords:** Endodontics, Hardness, Instrumentation, Simulate, Resin, Tooth

## Abstract

**INTRODUCTION:** We aimed to compare apical canal transportation of extracted teeth and two types of simulated resin blocks.

**MATERIALS AND METHODS:** Mesiobuccal root of extracted maxillary molars, high hardness simulated resin blocks (Knoop hardness=40) and low hardness simulated resin blocks (Knoop hardness=22) were prepared with K-files using step-back technique (n=15 canals in each group). Double exposure radiographic technique was used for extracted teeth. Simulated resin blocks were stabilized and scanned before and after preparation. Pre and post-preparation pictures were superimposed and apical transportation was measured. The data were analyzed statistically using ANOVA and Tukey HSD tests.

**RESULTS:** There was no significant difference in apical canal transportation between extracted teeth and high hardness resin blocks (P>0.05). Low hardness resin blocks showed more apical transportation than the other groups (P<0.05).

**CONCLUSION:** Under the conditions of this study, apical canal transportation for extracted teeth and high hardness simulated resin blocks were similar.

## INTRODUCTION

In the studies comparing the effects of instrumentation on the shape of the root canals, the standardization of variables is an important consideration. A definite problem in using extracted teeth in such studies is their inherent variability. Weine *et al.* were the first to notice this problem and instead used simulated root canals in clear casting resin which could be made to any predetermined size, shape or curvature as models for assessing the effects of root canal preparation (1). Such standardization would overcome the differences imposed by the multitude of variables inherent in extracted teeth, so that accurate evaluation of a particular technique or file type could be made (2). The major problem with resin blocks is their low hardness (3,4). Weine says Knoop hardness in resin blocks is equal to 22 kg/mm^2^ which is almost 40 kg/mm^2^ in natural teeth (5).

Different authors have used these models in their studies research (6-8). Ahmad have compared the effect of ultrasonic files on matched extracted teeth and resin blocks (2). The results of their study indicated that simulated canals in resin blocks are valid models for the assessment of root canal shapes following ultrasonic instrumentation. Also, a study carried out by Lim and Webber has shown that simulated root canals formed in clear casting resin were a valid experimental model for studying the shape of the prepared canal (9). In their studies, however, they did not mention the hardness effect on preparation manner in extracted teeth and simulated resin blocks.

This study was therefore undertaken to compare extracted teeth and resin blocks considering their Knoop hardness on apical canal transportation.

**Figure 1:**
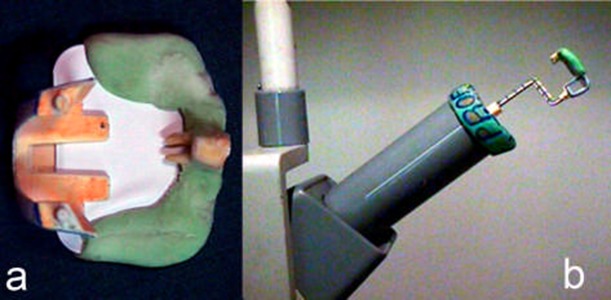
a) A radiograph mount for maintaining the tooth-film position constant, b) An Endo-ray attached to X-ray tube by acrylic resin

## MATERIALS AND METHODS

Three groups were selected: ***A)*** 15 high hardness simulated canals made of clear polyester resin (Farahani, Tehran, Iran) with standardized canal shape and Knoop hardness of 40 kg/mm^2^; ***B)*** 15 low hardness simulated canals made of clear polyester resin (Endo Training-Bloc, 0.02 Taper, Dentsply Maillefer, Ballaigues, Switzerland) with standardized canal shape and Knoop hardness of 22 kg/mm^2^ were used to assess instrumentation. The angle of curvature was 40^º^ in all simulated canals; and ***C)*** Extracted human maxillary molars were selected for this investigation. Radiographs were taken and teeth with open apices, canal calcification, external or internal resorption were excluded. Calculus and debris on the root surface of the remaining 15 teeth were removed using 7/8 Gracy curettes (Hu-Friedy, Chicago. IL, USA). Teeth were then stored in normal saline. Coronal access was achieved using diamond burs (D&Z, Berlin, Germany).

Fifteen mesiobuccal roots of which canals were freely accessible with a root-canal instrument size #10 up to the intact root tip, whose root-canal width near the apices was approximately compatible with size 10, and whose angle of curvature ranged between 35-45º were included. The determination of degree of curvature of mesiobuccal canals was based on Schneider method (10). The crown and palatal root were separated using a diamond disk so that all mesiobuccal canals had a working length of 16 mm. Double exposure method was used to measure canal transportation (11,12).

A radiographic mount was made to maintain constant tooth-film position (Figure 1a). The mount compromised a radiographic Endo-ray II paralleling device (Dentsply, Rinn Co., IL, USA) attached to X-ray tube by acrylic resin.

**Figure 2:**
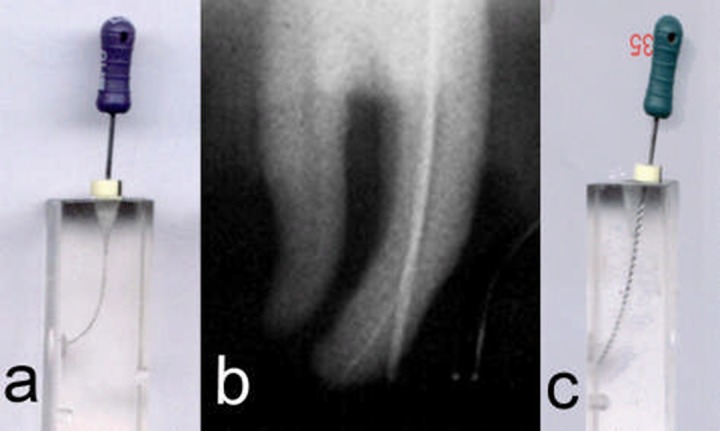
a) The scanned resin block with a #10 K-file, b) Apical transportation in MB canal, c) The scanned resin block with a #35 K-file

A Kodak Ultra-speed film (Kodak, Stuttgart, Germany) was attached to the bite block section of Endo-ray system and stabilized using acrylic resin (Figure 1b). The mesiobuccal root was placed over the X-ray film and covered with acrylic resin. Thus the long axis of the root canal was parallel and as close as possible to the surface of the film. The tube and central X-ray beam was aligned perpendicular to the root canal. Hence double exposure radiographic technique could be utilized.

Standardized radiographs were taken prior to instrumentation with an initial instrument size #10 inserted into the mesiobuccal canal. The simulated canals were also scanned before instrumentation with an instrument size #10 inserted in the canal (Figure 2a).

Hand instrumentation with stainless steel K-files (Maillefer, Ballaigues, Switzerland) was performed using a filing motion on all samples. A step-back method was used. The mesio-buccal canal of extracted teeth was prepared while the tooth was fixed to the film. All canals were sequentially prepared from file size #15-35 without pre-curving the instruments to the working length. After each instrument the canal was flushed with 5mL of 2.5% NaOCl solution using a plastic syringe (Supa, Tehran, Iran) with a gauge 27 irrigation tip. All procedures were performed by one experienced operator. At the end of canal preparation, mesiobuccal canals were radiographed with the final instrument inserted into the root canal. Pre- and postoperative radiographs were then scanned using CanoScan 4200F (Canon, Tokyo, Japan) (Figure 2b). The simulated resin blocks were also scanned with the final root canal instrument inserted in the canal (Figure 2c).

**Figure 3:**
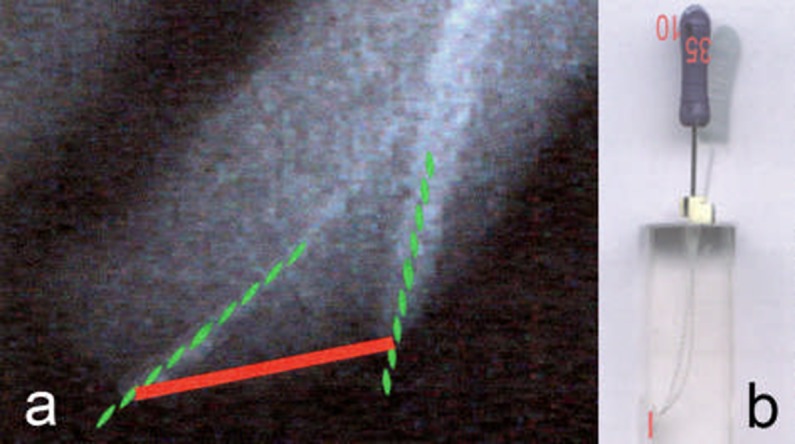
Measuring the apical transportation in a tooth (a) and a resin block (b)

In order to achieve a standardized position of the resin blocks on the scanner, a frame was made in which the resin blocks could be placed and repositioned in exactly the same position. The results of canal preparation were assessed using the Adobe Photoshop 8 software. Measurements were made on superimposed pre- and postoperative digitized images with ×10 magnification. The distance between the tip of the initial and final files were measured and the canal transportation was recorded (Figure 3a), (Figure 3b).

Data were evaluated statistically by analysis of variance (ANOVA) at a 0.05 significance level for global analysis, followed by a Post Hoc test.

## RESULTS

Analysis of data indicated that mean ± SD of transportation was 1.75±0.16 mm in extracted teeth, 1.8±0.19 mm in high hardness and 2.10±0.23 mm in low hardness resin blocks (Table 1). There was no statistical difference in apical transportation between extracted teeth and high hardness resin blocks (P=0.574). There was significant statistical difference in apical transportation between extracted teeth and low hardness resin blocks, as well as between high hardness and low hardness resin blocks (P<0.05).

## DISCUSSION

The purpose of this study was to compare the Knoop hardness of extracted teeth and simulated resin blocks on apical transportation.

For evaluating root canal preparation by different instruments, two more common experimental models were simulated root canals in clear resin blocks or root canals of extracted human teeth. Simulated root canals provide standardization of root canal diameter, length and curvature in terms of angle and radius (3). The credibility of resin blocks as an ideal experimental model for the analysis of endodontic preparation technique has been validated (1,13).

**Table1:** Mean (SD) values of apical transportation after preparation (mm)

Group	Mean (SD)	Min	Max
Natural teeth	1.76 (0.16)	1.48	2.08
High hardness blocks	1.83 (0.19)	1.60	2.20
Low hardness blocks	2.10 (0.23)	1.80	2.60

Most studies carried out on simulated resin blocks demonstrated that slight differences in hardness between dentin and the experimental resin can influence result of the clinical situation (3,4,14). Nevertheless, the use of simulated canals in resin blocks is the opportunity to standardize the research method and to exclude parameters that could influence the preparation outcome (4).

Extracted teeth have to be standardized to a degree i.e. have similar apical patency, compatibility of apex to a specified instrument size and angle of curvature. The determination of curvature of mesiobuccal root canals was based on Schneider’s study (10).

Sepic *et al.* (15) and also Wu and Wesselink (16) demonstrated that step-back technique resulted in more apical transportation than balanced-force technique. Therefore, to have a better inspection of transportation, step-back technique was used in this study.

Tharuni *et al.* (6) demonstrated that K-files *i.e.* stainless steel hand instruments, result in more apical transportation in comparison with NiTi Lightspeed in simulated resin blocks. Stainless steel K-files were used during the step back technique without previous curvatures, so that the maximum possible apical transportation could be measured.

When comparing the shaping ability of root canal instruments, it is important to have a similar apical diameter (17). The minimum apical preparation diameter should be a size 35 for maximum cleanliness of the canal (18).

This was carried out in this study. A radiographic platform was fabricated that allowed for accurate pre and post-operative radiographs to be taken on the same film. This method was also used by Luiten and Lumley (11) and Kavanagh *et al.* (12). To measure apical transportation in simulated resin blocks, a frame was made and blocks were scanned with canal instrument size 10 before instrumentation and to a canal instrument size #35, post instrumentation.

The difference in apical transportation between extracted teeth and high hardness resin blocks was not statistically significant concurring with previous studies (2). They found no difference in apical transportation between resin blocks and extracted teeth. However the type of resin, its hardness and the manufacturer are not mentioned in their study.

The present data showed that resin blocks with lower hardness (Knoop=22) had more transportation than extracted teeth and resin blocks which had high hardness (Knoop=40). The fact that high hardness resin blocks and extracted teeth showed similar transportation agrees with the results of Ahmad (2). In their research on simulated resin blocks and extracted teeth, they found no significant difference in transportation using ultrasonic and hand files. However the resin type, hardness, and manufacturer were not mentioned.

Since high hardness simulated resin blocks and extracted teeth have similar hardness; Knoop hardness may have influenced the instrument manner during canal preparation. Therefore, resin hardness can play a role in the results of studies carried out on simulated resin blocks.

The question remains as to how critical the hardness effect is in extrapolating the results of simulated resin block studies to the clinical situation.

## CONCLUSION

Within the limitation of this *in vitro* study high hardness simulated resin blocks and extracted teeth showed similar apical transportation because of their comparable Knoop hardness. Low hardness resin blocks displayed more apical transportation than extracted teeth as well as high hardness simulated resin blocks.
